# T cell receptor repertoire profiling predicts the prognosis of HBV‐associated hepatocellular carcinoma

**DOI:** 10.1002/cam4.1610

**Published:** 2018-06-26

**Authors:** Kai‐Rong Lin, Fei‐Wen Deng, Ya‐Bin Jin, Xiang‐Ping Chen, Ying‐Ming Pan, Jin‐Huan Cui, Zhi‐Xuan You, Huan‐Wei Chen, Wei Luo

**Affiliations:** ^1^ Clinical Research Institute Foshan Hospital Sun Yat‐sen University Foshan China; ^2^ Department of Hepatobiliary Surgery Foshan Hospital Sun Yat‐sen University Foshan China; ^3^ Zhixin Middle School Guangzhou China

**Keywords:** hepatocellular carcinoma, high throughput sequencing, infiltrating T lymphocyte, prognosis, TCR repertoire

## Abstract

Tumor‐infiltrating T cell repertoire has been demonstrated to be closely associated with anti‐tumor immune response. However, the relationship between T cell repertoire in tumor tissue and prognosis has never been reported in Hepatocellular carcinoma (HCC). We performed the high‐throughput T cell receptor (TCR) sequencing to systematically characterize the infiltrating T cell repertoires of tumor and matched adjacent normal tissues from 23 HBV‐associated HCC patients. Significant differences on usage frequencies of some Vβ, Jβ, and Vβ‐Jβ paired genes have been found between the 2 groups of tissue samples, but no significant difference of TCR repertoire diversity could be found. Interestingly, the similarity of TCR repertoires between paired samples or the TNM stage alone could not be helpful to evaluate the prognosis of patients very well, but their combination could serve as an efficient prognostic indicator that the patients with early stage and high similarity showed a better prognosis. This is the first attempt to assess the potential value of TCR repertoire in HCC prognosis, and our findings could serve as a complement for the characterization of TCR repertoire in HCC.

## 1 INTRODUCTION

Hepatocellular carcinoma (HCC) is one of the most common malignancies of the liver in the world.^1^ It is the third most frequent cause of cancer‐related mortality, with over 500 000 people affected. Most cases of HCC are secondary to chronic liver diseases, such as hepatitis B virus (HBV) or hepatitis C virus (HCV) infection, hepatic cirrhosis or primary biliary cholangitis.^2^, ^3^, ^4^ Approximately 80% of HCC are associated with chronic HBV infection.^5^ It has been proven that HBV can cause the immune attacks of liver due to the immune‐mediated mechanisms, leading to the development of hepatocyte inflammation, necrosis, proliferation, and finally oncogenic transformation.^6^ The development and prognosis of HBV‐associated HCC are closely related to individual immunity, especially cellular immune response mediated by T lymphocyte.^7^

Infiltrating T lymphocytes are the major effector cells in tumor lesion, which play an important role in the occurrence and evolution of tumor. It have been demonstrated that infiltrating T lymphocytes are closely associated with tumor development and clinical outcome in various tumor types.^8^, ^9^, ^10^ T cells are selectively activated and undergoing a massive expansion via specific recognition of tumor neoantigens. T cells repertoires have been identified to coevolve with the spectrum of neoantigens over time, which result in a characteristic manifestation of T cells repertoire specific for malignant tumor in lesion.^11^, ^12^

It has been hypothesized that in different immune‐mediated chronic liver diseases, a disease‐associated antigenic epitope profile drives the recruitment and expansion of T cells to form a distinctive T‐cell repertoire within the liver. By high throughput TCR sequencing of T‐cell repertoires, differential TCR signatures had been identified in primary biliary cirrhosis, primary sclerosing cholangitis, and alcoholic liver disease, which represented an imprint of distinctive antigenic repertoires in these chronic liver diseases.^13^ Similarly, significant differences of T‐cell repertoires among HCC, intrahepatic cholangiocarcinoma, and mixed hepatocellular and cholangiocellular carcinoma have also been demonstrated.^14^ Besides, the T‐cell repertoires in tumor and matched adjacent nontumor tissues from HBV‐associated HCC patients have also shown a significant difference, suggesting a distinct T cell immune microenvironment.^15^ Even in distinct regions of the same tumor, there were different tumor infiltrating T cell clones existed, reflected by the percentage of TCR sequences, regional frequencies of each clone and their diversity.^16^ Although tumor‐infiltrating T‐cell repertoire was reported to be closely associated with host antitumor immune response, the relation between TCR repertoire and cancer prognosis has never been reported in HCC patients.

In this study, the T‐cell repertoires in tumor and adjacent normal tissues from HBV‐associated HCC patients were analyzed by high‐throughput TCR sequencing. Except for the characterization and comparisons of the diversity and similarity of T‐cell repertoires, our work also focused on the potential value of TCR repertoire in prognosis of HBV‐associated HCC patients.

## 2 MATERIALS AND METHODS

### 2.1 Patients and sample collection

Tumor and adjacent normal tissue specimens were collected from 23 patients diagnosed with primary HCC by histopathology and treated with surgical resection at Foshan Hospital of Sun Yat‐sen University (Guangdong, China) from May 2013 to Jan 2017. None of the 23 patients had received chemotherapy or radiotherapy, or had other immune‐related diseases, such as infectious diseases, autoimmunity diseases, and other tumors.

All the tissue samples were confirmed independently by 3 pathologists with extensive clinical experience. Considering the spatial heterogeneity of the tumor tissues, 8 different spatial sites of each tissue sample were collected and mixed together to add TRIzol (Invitrogen, USA). The tissue lysates were immediately stored at liquid nitrogen until further processing. This study was conducted in accordance with the Declaration of Helsinki and approved by the Ethics Committee of Foshan Hospital of Sun Yat‐sen University. All patients in this study provided written informed consents for their participation.

### 2.2 High‐throughput sequencing

Total RNA was extracted from 1 mL of tissue lysate using total RNA Kit (OMEGA), according to the manufacturer's instructions. Total RNA was reverse transcribed into cDNA by using SMARTer PCR cDNA synthesis kit (Clontech, USA).^17^ Seminested PCR amplification was conducted to prepare TCR library (the SMARTer^®^ RACE 5′/3′ Kit, Clontech). Briefly, for the first‐round reaction, cDNA was amplified with 0.2 mL of Advantage 2 polymerase mix (Clontech, USA), Nested Universal Primer (NUP, Clontech, 5′‐AAGCAGTGGTATCAACGCAGAGT‐3′), and 3′‐TCR β outer primer (5′‐AGATCTCTGCTTCTGATGGCT‐3′) according to the following cycling conditions: 94°C for 3 min, then carried out with 35 cycles of denaturing at 94°C for 15 seconds, annealing at 58°C for 30 seconds. The first‐round PCR products purified by gel extraction kit (QIAGEN, German) were used as template for the second amplification with 3′‐TCR β inner primer (5′‐TGGCTCAAACACAGCGACCT‐3′) using the same PCR conditions as the first round. The 3′‐TCR β outer and inner primers were both homologous to the 3′‐TCR β constant regions, which has been proven to be reliable and valid primers for TCR specific amplification in our previous studies.^18^, ^19^, ^20^ The second‐round PCR products were then recycled and purified by gel extraction kit. Finally, 1.5 μg objective product per sample was used as a TCR sequencing library to perform the high throughput sequencing on the Hiseq platform. The read length of sequencing was 150 bp.

In addition, the sequencing technology platform used in our study had been proven to be feasible and repeatable by a technical duplicate test. A separate sample was amplified by PCR and sequenced in duplicate for comparative analysis. Both V‐J pairs and clonotypes obtained from the 2 technical duplicates showed in Figure S1A,B, with strong correlations.

### 2.3 TCR sequence analysis

The sequencing data were stored in FASTQ format. Firstly, the low‐quality sequences were filtered out and the remainders were reserved for further analysis. BLAT software was used to find TCR Vβ, Jβ, and Cβ genes in each sequence at the TCR reference genome downloaded from IMGT/GeneDB database.^21^ Those sequences containing Vβ, Jβ, and Cβ gene segments were extracted and further translated into aa sequences. Finally, the sequences without terminator were selected as the productive aa sequences for further analysis.

### 2.4 Statistical analysis

Wilcoxon signed rank test, paired *t*‐test, Mann‐Whitney test and Student's *t*‐test were used to compared different groups if appropriate; two‐sided *P* values reported were considered significant when *P* < .05. PFS were calculated from the date of diagnosis of HCC and assessed by life‐table analysis. Kaplan‐Meier analysis and Log‐rank test were used to compare the difference of PFS between groups. The receiver operating characteristic (ROC) curve was used to evaluate the efficiency of prognostic indicators for HBV‐associated HCC. The area under curve (AUC) was calculated by the Hanley and McNeil method. Graphpad Prism (version 5.1) and SPSS20.0 software were used to analyze and acquired the representative images.

## 3 RESULTS

### 3.1 Patient characteristics

The demographic and clinical characteristics of 23 patients with primary HBV‐associated HCC included in this study were listed in Table 1. All of patients were male, and the median age was 44 years (range: 29‐59 years). According to the union for international cancer control (UICC, 2002) staging system, a total of 8(34.8%) patients were stage I, 7 (30.4%) patients were stage II, and 8 (34.8%) patients were stage III. Among them, there were 8 (34.8%) patients with recurrence or metastasis during the follow‐up.

**Table 1:** The demographic and clinical characteristics of 23 patients with primary HCC

Characteristics	No. (%)
Age at diagnosis (in y)	
Median (Range)	44 (29‐59)
Gender	
Male	23 (100%)
Tumor (T) stage	
T1	8 (34.8%)
T2	7 (30.4%)
T3(A + B)	8 (34.8%)
Lymphoid Nodal (N) status	
N0	23 (100%)
Distant metastasis (M) status	
M0	23 (100%)
TNM stage	
I	8 (34.8%)
II	7 (30.4%)
III (A + B)	8 (34.8%)
Progression in 2 y	
No	15 (65.2%)
Yes	8 (34.8%)
HBV	
Positive	23 (100%)

### 3.2 High‐throughput sequencing of TCR repertoire in tumor and adjacent normal tissues

The sequence profiles of TCR β chain in tumors or paired adjacent normal tissues from 23 HBV‐associated HCC patients had been obtained and shown in Table S1. A total of 29 103 540 productive amino acid (aa) sequences were obtained from 23 paired samples, with an average of 632 685 per sample. The average number of productive unique aa sequences was 10 081 per sample. Sixty‐five V gene segments and thirteen J gene segments had been identified in most of samples, which combined into a total of 780 distinct V‐J pairs.

**Figure 1:**
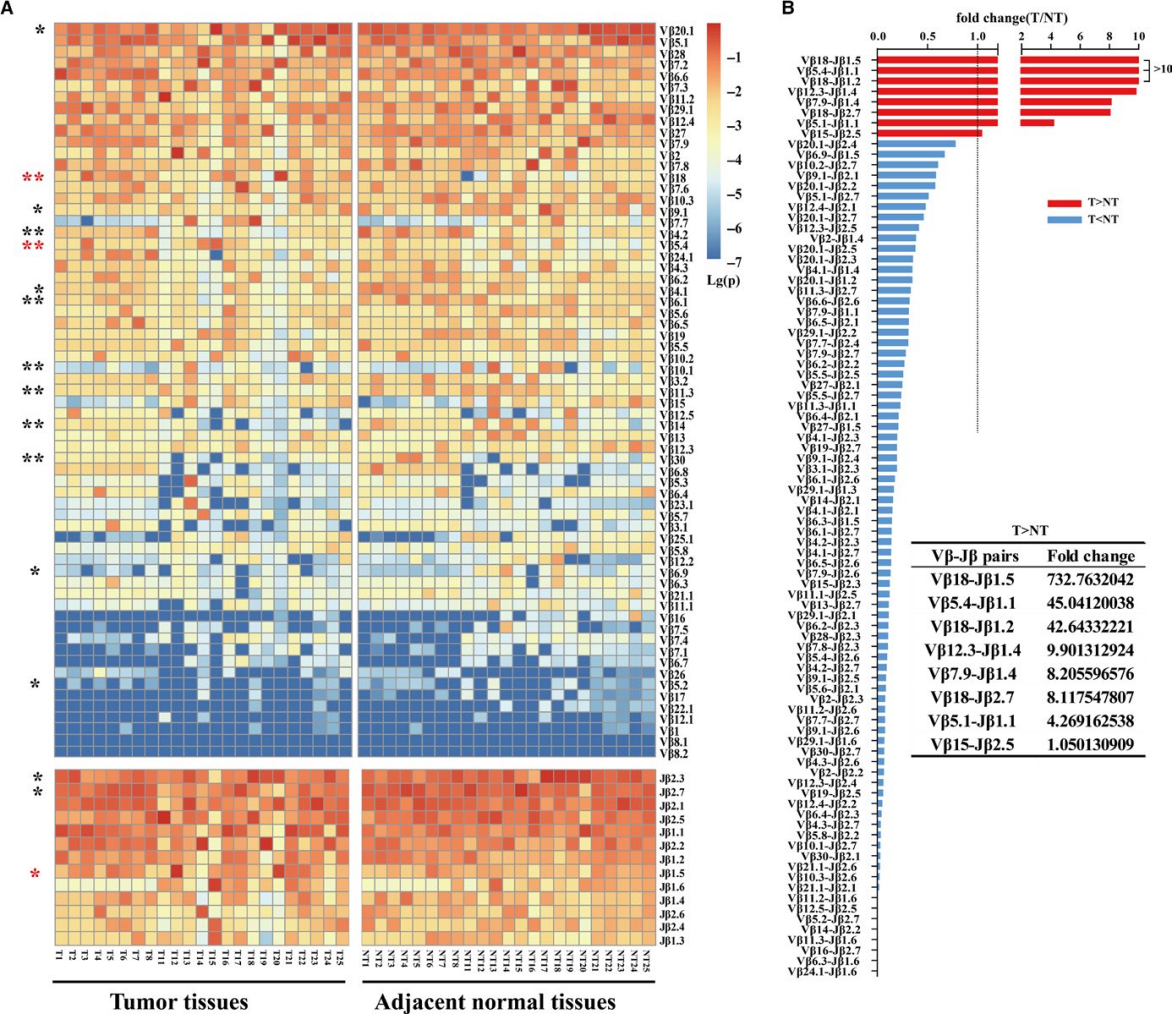
The usage of Vβ, Jβ, and Vβ‐Jβ paired genes in tumor tissues and paired adjacent normal tissues. A, Heat map of the usage frequency of Vβ and Jβ genes. *P <* *.05 and **P <* *.01 by two‐tailed paired *t*‐tests. Several Vβ or Jβ usage frequencies were significantly higher (red star) or lower (black star) in tumor tissue than paired adjacent normal tissue. B, The Vβ‐Jβ paired genes with significantly difference on usage frequency between paired tissues. Fold change was calculated by the usage frequency in tumor tissue divided by the usage frequency in paired adjacent normal tissue. T, tumor tissue; NT, paired adjacent normal tissue

The usage frequency of TCR Vβ and Jβ gene in each sample were respectively delineated in Figure 1A. There were obvious Vβ/Jβ gene usage biases in tissue samples. The top 5 frequent genes were Vβ20.1 (average of 16.10%), Vβ5.1 (12.64%), Vβ28 (6.19%), Vβ7.2 (5.18%), and Vβ6.6 (4.74%), as well as Jβ2.3 (18.10%), Jβ2.7 (15.78%), Jβ2.1 (15.07%), Jβ2.5 (11.58%), and Jβ1.1 (11.51%). Notably, Vβ20.1, Vβ5.1 and Jβ2.7, Jβ2.1 were also the most frequent gene segments in other studies of tumors and healthy subjects.^15^

Comparative analysis of the Vβ, Jβ, and Vβ‐Jβ paired gene usage in TCR repertoires between the tumors and paired adjacent normal tissues were performed to further reveal the property of infiltrating T cells. Overall, the usage pattern of Vβ or Jβ genes in tumor tissues was grossly similar to matched adjacent normal tissues, but the usage frequency of 13Vβ genes and 3Jβ genes were significantly different between tumor and adjacent normal tissues (*P *<* *.01).

Some Vβ or Jβ genes used in tumor tissues were higher than adjacent normal tissues, while others were the opposite (Figure 1A). Besides, 88 distinct Vβ‐Jβ pairs showed significant usage frequency differences between the 2 tissue groups (*P *<* *.01). Only 8 Vβ‐Jβ pairs showed higher frequency usage in tumor tissues (Figure 1B).

### 3.3 No difference of TCR repertoire diversity between tumor and adjacent normal tissues

We calculated the Clonality index (1‐ (Shannon's entropy)/log2 (number of productive aa unique sequences)) and the U/T index (the number of productive unique aa sequences/the number of total productive aa sequences) to estimate the diversity of T cell clones in each sample, which were independent of sample sequencing depth.^13^, ^22^, ^23^

**Figure 2:**
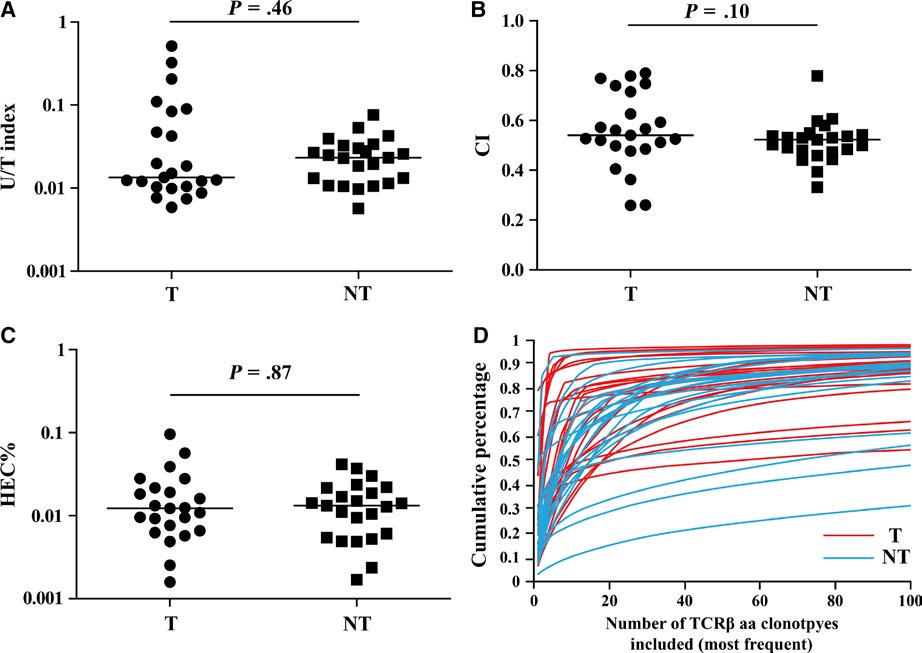
Comparison of the TCR repertoire diversity indexes between tumor tissues and paired adjacent normal tissues. A, U/T index. B, Clonality index. C, The percentage of highly expanded clones. D, The cumulative percentage of the most 100 frequent TCRβ sequences in each sample. Differences between groups were compared using Wilcoxon matched pairs test. The horizontal line means the median. T, tumor tissue; NT, paired adjacent normal tissue

The result showed that the TCR repertoire diversity among tumor or adjacent normal tissue samples from different patients were very different (Table S1), which might indicate that the richness of infiltrating T cells were different among HCC patients. There were no significant differences of TCR repertoire diversity between tumor and paired adjacent normal tissues by paired *t* test (U/T index, *P *=* *.46, Figure 2A; Clonality index, *P *=* *.10, Figure 2B). Furthermore, we assessed the frequency distribution of TCR repertoire by the ratio of highly expanded clones (HEC, with frequencies >0.1%) and the cumulative percentage of the most 100 frequent TCR aa sequences. Consistent with the diversity indexes, the 2 frequency distribution indexes showed no significant difference between tumor and adjacent normal tissues (Figure 2C,D).

### 3.4 No association between intratumoral TCR repertoire diversity and clinical TNM stage or prognosis

**Figure 3:**
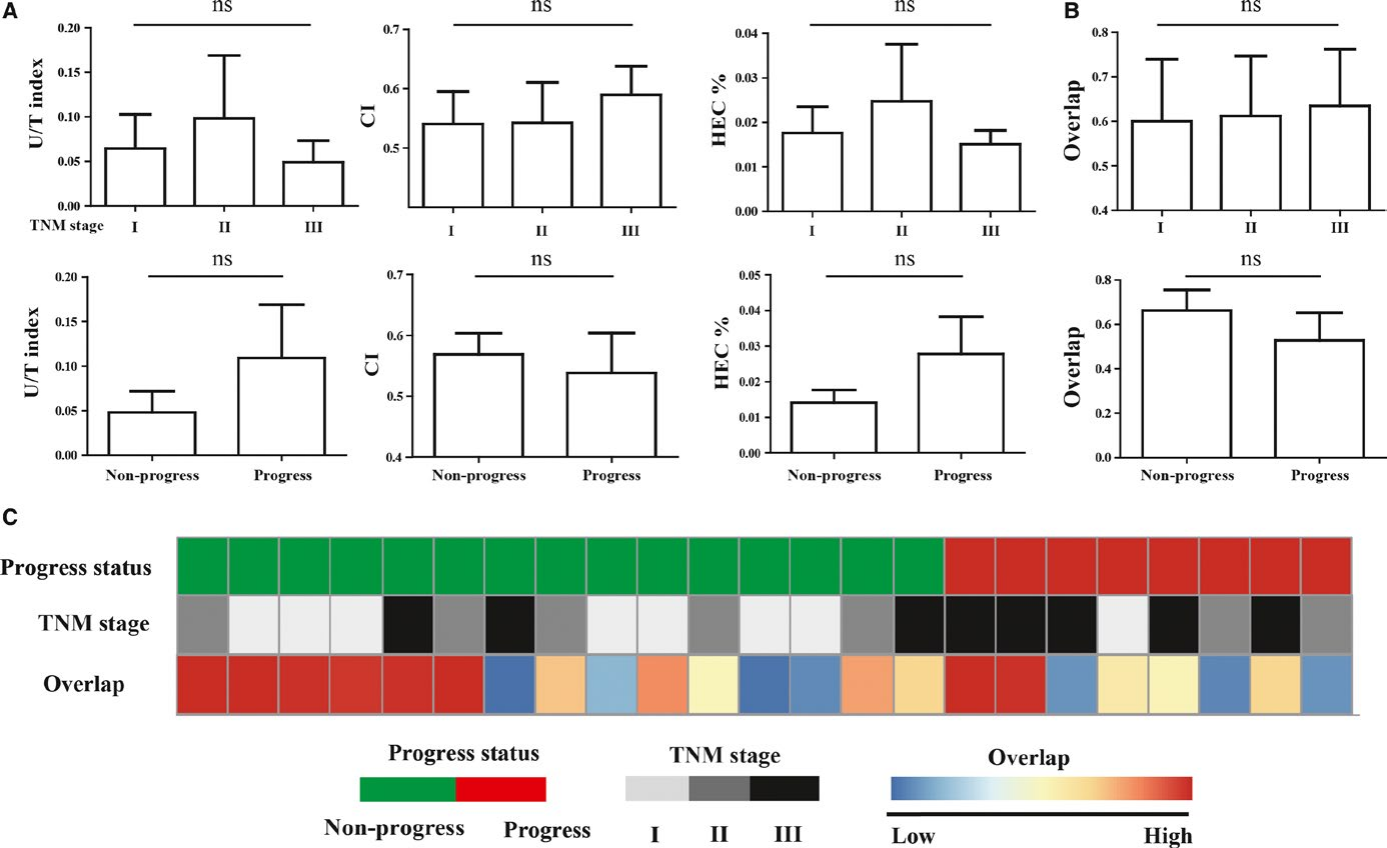
Analysis of the relation between TCR repertoire and clinical data. A, Comparison of TCR repertoire diversity in tumor tissues among different groups. B, Comparison of TCR repertoire similarity of paired samples among different groups. TCR repertoire overlap between paired samples was calculated by overlap. C, Heat map associations of TCR repertoire similarity and clinical information in 23 patients. TNM stages (I, n = 8; II, n = 7; III, n = 8). Disease progress group (n = 8) and non‐progress group (n = 15). ns indicates not significant difference

We further investigated the association between intratumoral TCR repertoire diversity and clinical TNM stage or prognosis. There were no significant differences of the U/T index, Clonality index or HEC ratio among 3 different clinical stage groups (stage I, n = 8; stage II, n = 7; stage III, n = 8), as well as between disease progress group (tumor recurrence or metastasis, n = 15) and disease nonprogress group (n = 8). The TCR repertoire diversity in tumor tissue from patients in disease progress group was slightly higher (Figure 3A).

### 3.5 Similarity of TCR repertoire between tumor and adjacent normal tissues was associated with prognosis

The TCR repertoire overlap was always used to evaluate the similarity of TCR repertoires between samples, calculated as the total number of shared productive aa sequences divided by the sum of productive aa sequences detected in 2 samples.^24^ We quantified the similarity of TCR repertoire between tumor and adjacent normal tissues by overlap, and investigated it's relation with the TNM stage or prognosis. The overlap of paired samples from 23 HBV‐associated HCC patients ranged from 0.0765 to 0.987, with the median of 0.685. We did not find any relation between the overlap and TNM stage. Notably, the overlap of paired samples from patients with disease progression was slightly lower than those from patients without disease progression (Figure 3B,C).

**Figure 4:**
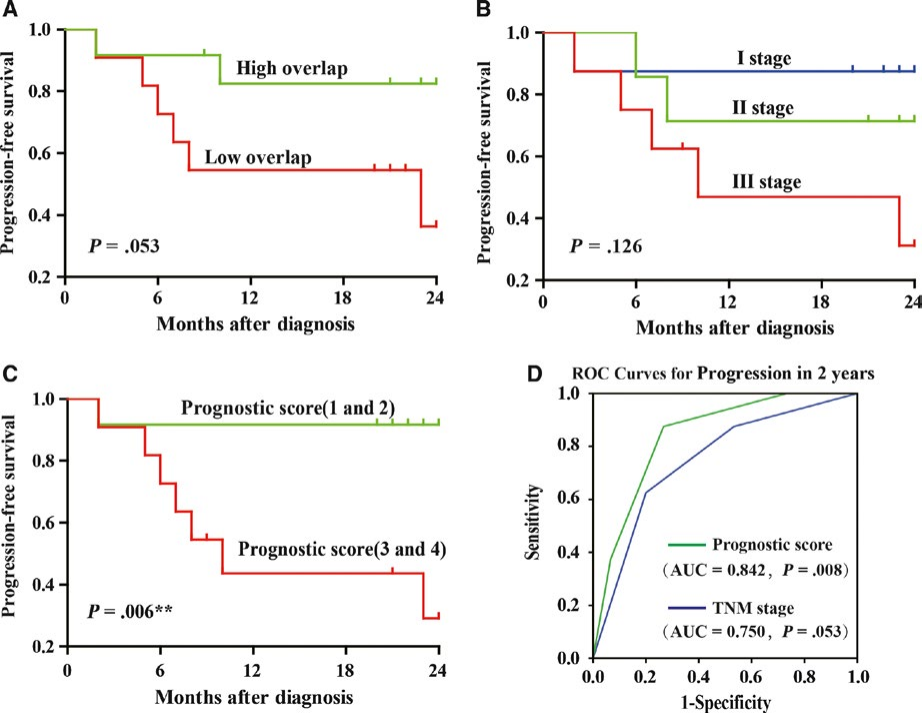
Analysis of the relation between TCR repertoire similarity and clinical data. A, The PFS curves of the high similarity group (n = 12) and the low similarity group (n = 11). B, The PFS curves of the different TNM stage groups (I, n = 8; II, n = 7; III, n = 8). C, The PFS curves of the 1 and 2 prognostic score group (n = 12) and the 3 and 4 prognostic score group (n = 11). D, ROC analysis for the prognostic score and the AUC value was 0.842 relative to 0.750 for TNM stage

Using the median of overlap as a cut‐off, 23 patients were divided into high overlap group (n = 12) and low overlap group (n = 11). We found that the progression‐free survivals (PFS) of patients in the high overlap group were higher than those in the low overlap group but without significant difference (*P *=* *.053; Figure 4A). Similarly, the PFS of patients did not significantly differed in varied TNM stage groups (*P *=* *.126; Figure 4B).

We expected that the combination of these 2 parameters might obtain a better prognostic indicator for HCC patients. So, the prognostic score of each patient was calculated based on overlap and TNM stage. Patient with stage I would obtain 1 score, with stage II would obtain 2 score, and with stage III would obtain 3 score. In addition, patient in low overlap group would obtain extra 1 score, while patient in high overlap group do not. Interestingly, the result showed that the PFS of patients with lower prognostic score was significantly higher than those with higher prognostic score (*P *=* *.006; Figure 4C). The prognostic score displayed an AUC value of 0.842 by ROC curve analysis, while TNM stage only displayed an AUC value of 0.750 (Figure 4D), suggesting the combination of overlap and TNM stage has a better prognostic effect.

## 4 DISCUSSION

An in‐depth comprehension on TCR repertoires of infiltrating T cells in patients with primary HBV‐associated HCC has been presented in this study. When compared the TCR repertoire diversity between tumor tissues and matched adjacent normal tissues, we did not find significant difference between paired tissues. This result was inconsistent with a previous study on another cohort of HBV‐associated HCC patients, which found that TCR repertoire diversity in tumor tissues is higher than adjacent nontumor tissues.^15^ Besides, the Vβ and Jβ genes with differential usage in our study were also inconsistent with this study, except for Vβ5.4 and Vβ18 genes that were higher in tumor tissues than in adjacent normal tissues in both studies.^15^ The intrinsic different T cell infiltration in tumor and adjacent normal tissues from distinct individuals may serve as a major reason for these inconsistent results.

However, it was worth mentioning that there were 2 advantage of TCR repertoire sequencing and assessing methods in our study. Firstly, we performed the TCR sequencing using increased sequencing reads and read length, which could contribute to obtaining a more reliable data and conclusion. Secondly, we used the U/T index and the Clonality index rather than the number of productive unique aa sequences or Shannon diversity index to assess the diversity of TCR repertoire, as the former 2 indexes could better correct the impact of uneven reads amount.^13^, ^22^, ^24^ Using the different sequencing depth or the different diversity indicators might be another reason for the inconsistent results. Our study could serve as a complement and these findings need to be corroborated in a larger cohort.

When compared the TCR repertoire similarity between tumor tissues and matched adjacent normal tissues, we found that there were obviously interindividual differences. What's interesting is that the similarity of TCR repertoires between paired samples or the TNM stage alone could not be helpful to evaluate the prognosis of patients very well, but the combination of TCR repertoire similarity and TNM stage showed to be a better prognostic indicator for HCC patients. The relation between the TCR repertoire and clinical prognosis has never been reported in HCC patients previously.

In a previous study on gastric cancer, it has been reported that the similarities of TCR repertoires between tumor and adjacent normal tissues were gradually decreasing during malignant progression.^25^ Theoretically, malignant tumor progression was associated with the increase of tumor mutation load and the generation of immunogenic neoantigens,^26^, ^27^ forming an extremely different local microenvironment in tumor tissue. T cell repertoire would correspondingly evolve with the change of neoantigens, and gradually deviate from the T cell repertoire in adjacent normal tissues. Therefore, higher similarity of TCR repertoire might suggest lower tumor mutation load, which was associated with a better prognosis.

In conclusion, we have systematically characterized the infiltrating T cell repertoires of tumor and adjacent normal tissues from HBV‐associated HCC patients. We did not find significant difference of the TCR repertoire diversity between paired tissues, but found higher similarity of TCR repertoire between paired tissues was associated with better prognosis. More interesting, the combination of TCR repertoire similarity and TNM stage showed to be a better prognostic indicator. This is the first attempt to assess the potential value of TCR repertoire in HCC prognosis.

## CONFLICT OF INTEREST

The authors declare that they have no competing interests.
