# Supplementary figures and images for: T cell receptor repertoire profiling predicts the prognosis of HBV‐associated hepatocellular carcinoma
Source: Cancer Med. 2018 Jun 26;7(8):3755–62. doi: 10.1002/cam4.1610 (PMC6089190; doi:10.1002/cam4.1610)

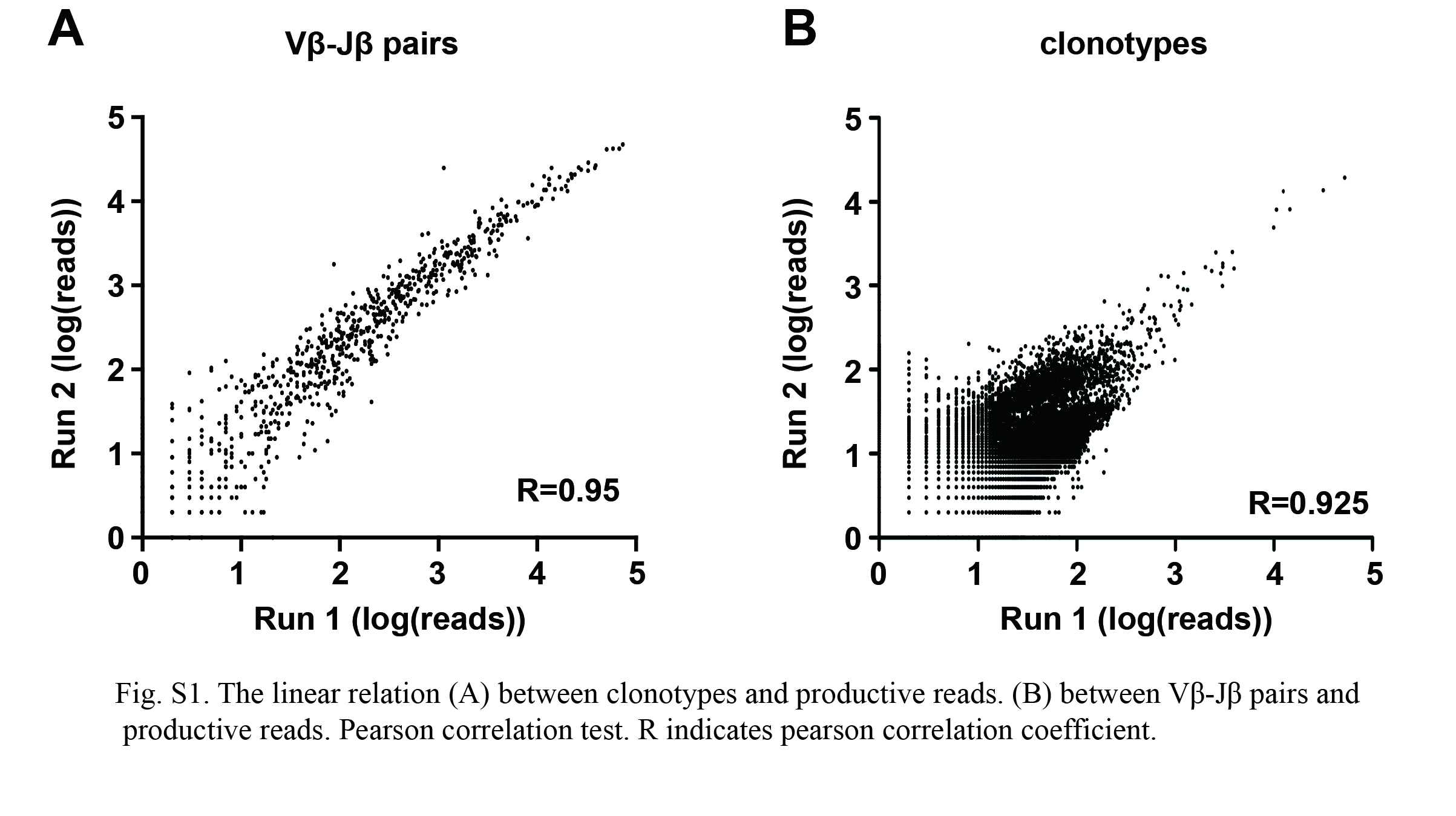

Supplement: Supplementary file 1 [file CAM4-7-3755-s001.tif]
